# Long – acting injectable aripiprazole in patients with psychosis is associated with improved quality of life, better general clinical outcome and fewer hospitalizations

**DOI:** 10.1192/j.eurpsy.2024.1516

**Published:** 2024-08-27

**Authors:** E. I. Koiliari, I. Mouzas, G. Alevizopoulos, O. Lesch, H. Walter, E. L. Pasparakis

**Affiliations:** ^1^Laboratory of Alcohology, Department of Pathology, Medical School of Crete, University of Crete, Herakleion of Crete; ^2^Department of Psychiatry, General Hospital of Agios Nikolaos, Agios Nikolaos of Lasithi; ^3^Department of Psychiatry, Agioi Anargyroi Hospital, National and Kapodistrian University of Athens, Athens, Greece; ^4^Department of Social Psychiatry, Medical University of Vienna, Vienna, Austria

## Abstract

**Introduction:**

Aripiprazole, a D2 receptor partial agonist is suggested to enhance Prefrontal Cortex (PFC) dopamine functioning resulting to an improvement of working memory and GABA transmission related to social functioning. The LAI form of the medication is documented to improve the long-term adherence of the patients resulting in a better assessment of the effects of the drug on behavioral parameters that require a longer time to evaluate.

**Objectives:**

Hypothesis testing: “Aripiprazole LAI antipsychotic treatment is associated with i) reduced hospitalizations, ii) improved quality of life and iii) patient functioning”.

**Methods:**

65 patients participated (Male to Female ratio corresponds to 2:1). 44 of them, the community population manifested psychosis (23 schizophrenia and 21 patients bipolar disorder with psychotic features). The median age was 41 years. 31.8% had dual diagnosis of psychosis and alcohol use disorders, while 25% had dual diagnosis of psychosis and Cannabis Use disorder. 77.3% were on aripiprazole LAI. 21 patients with BD I were prisoners at the Penitentiary of Neapolis of Lasithi of Crete. Median age was 36 years (all men). 90.5% had comorbidity of bipolar disorder type I (BD-I) and alcohol use disorders. 95.2% had comorbidity of BD – I and Cannabis Use Disorder. All were medicated by aripiprazole LAI 400mg/month. For the evaluation of our hypotheses the instruments WHOQOL-BREF questionnaire and the CGI-S scale were used. The quality of life, functionality, and number of hospitalizations were compared in each patient, before the initiation of the LAI medication and during the active treatment period. The minimum of follow-up period was 6 months.

**Results:**

In 44 patients (in community) hospitalizations decreased statistically significantly from 1.3±1.9 to 0.1±0.4 (Paired Samples Wilcoxon Signed Rank Test p-value<0.001). The CGI-S score decreased statistically significantly from 6.0 ±0.8 to 4.0±1.1 (Paired Samples Wilcoxon Signed Rank Test p-value<0.001). The score of the WHOQOL-BREF scale increased statistically significantly from 0.5 ± 0.5, to 2.9 ± 0.8 (Paired Samples Wilcoxon Signed Rank Test p-value<0.001). For the group of 21 patients (imprisoned) hospitalizations decreased from 0.6 ± 1.8 to 0.0 ± 0.0 (Paired Samples Wilcoxon Signed Rank Test p-value=0.066). The CGI-S score decreased statistically significantly from 5.3 ± 0.8 to 3.2 ± 1.3 (Paired Samples Wilcoxon Signed Rank Test p-value<0.001). The quality-of-life scale score increased statistically significantly from 0.9 ±0.6 to 3.09±0.7 (Paired Samples Wilcoxon Signed Rank Test p-value<0.001).

**Conclusions:**

Aripiprazole LAI significantly improves the quality of life and functionality of patients with psychosis. We suggest that the improvement might be related to the beneficial effects of the molecule on the Prefrontal Cortex (PFC).

**Disclosure of Interest:**

None Declared

